# Validation of a Salivary RNA Test for Childhood Autism Spectrum Disorder

**DOI:** 10.3389/fgene.2018.00534

**Published:** 2018-11-09

**Authors:** Steven D. Hicks, Alexander T. Rajan, Kayla E. Wagner, Sarah Barns, Randall L. Carpenter, Frank A. Middleton

**Affiliations:** ^1^Department of Pediatrics, Penn State College of Medicine, Hershey, PA, United States; ^2^Quadrant Biosciences, Inc., Syracuse, NY, United States; ^3^Departments of Neuroscience and Physiology, State University of New York Upstate Medical University, Syracuse, NY, United States; ^4^Departments of Psychiatry, Biochemistry and Molecular Biology, State University of New York Upstate Medical University, Syracuse, NY, United States; ^5^Department of Pediatrics, State University of New York Upstate Medical University, Syracuse, NY, United States

**Keywords:** autism, machine learning, RNA, microRNA, transcriptome, epigenetics, diagnosis, screening

## Abstract

**Background:** The diagnosis of autism spectrum disorder (ASD) relies on behavioral assessment. Efforts to define biomarkers of ASD have not resulted in an objective, reliable test. Studies of RNA levels in ASD have demonstrated potential utility, but have been limited by a focus on single RNA types, small sample sizes, and lack of developmental delay controls. We hypothesized that a saliva-based poly-“omic” RNA panel could objectively distinguish children with ASD from their neurotypical peers and children with non-ASD developmental delay.

**Methods:** This multi-center cross-sectional study included 456 children, ages 19–83 months. Children were either neurotypical (*n* = 134) or had a diagnosis of ASD (*n* = 238), or non-ASD developmental delay (*n* = 84). Comprehensive human and microbial RNA abundance was measured in the saliva of all participants using unbiased next generation sequencing. Prior to analysis, the sample was randomly divided into a training set (82% of subjects) and an independent validation test set (18% of subjects). The training set was used to develop an RNA-based algorithm that distinguished ASD and non-ASD children. The validation set was not used in model development (feature selection or training) but served only to validate empirical accuracy.

**Results:** In the training set (*n* = 372; mean age 51 months; 75% male; 51% ASD), a set of 32 RNA features (controlled for demographic and medical characteristics), identified ASD status with a cross-validated area under the curve (AUC) of 0.87 (95% CI: 0.86–0.88). In the completely separate validation test set (*n* = 84; mean age 50 months; 85% male; 60% ASD), the algorithm maintained an AUC of 0.88 (82% sensitivity and 88% specificity). Notably, the RNA features were implicated in physiologic processes related to ASD (axon guidance, neurotrophic signaling).

**Conclusion:** Salivary poly-omic RNA measurement represents a novel, non-invasive approach that can accurately identify children with ASD. This technology could improve the specificity of referrals for ASD evaluation or provide objective support for ASD diagnoses.

## Introduction

Children with autism spectrum disorder (ASD) are characterized by social/communication deficits and restricted/repetitive behaviors, but display marked variation at the genetic, phenotypic, and functional levels (Geschwind, 2008). Screening for ASD typically relies on the modified checklist for autism in toddlers – revised (MCHAT-R), a parent-based survey with a positive predictive value less than 20% (Siu et al., 2016). In 2017, the U.S. Preventive Services Task Force determined that insufficient evidence existed to recommend continued ASD screening. Non-specific screening tools may lead to over-referral, contributing to wait times that exceed 12 months for diagnostic evaluation using the Diagnostic and Statistics Manual (DSM)-5 criteria (Bisgaier et al., 2011). This wait can delay initiation of critical intervention services at a time of marked brain development. Addition of a rapid, accurate, objective adjunct measure could improve care for children with ASD.

Efforts to identify ASD biomarkers have yielded much information about the biologic basis of ASD. For instance, children with ASD are typified by hyperserotonemia (Chamberlain and Herman, 1990), elevated oxidative stress markers (Chauhan and Chauhan, 2006), and alterations in immune factors that can lead to neuroinflammation (Corbett et al., 2006; Goines and Van de Water, 2010; Onore et al., 2012). Such disturbances may facilitate disruptions in neuropeptides, including glutamate, GABA (El-Ansary and Al-Ayadhi, 2014), and brain derived neurotrophic factor (BDNF) (Connolly et al., 2006). Evidence suggests that environmental risk factors also play a role in ASD development (Hertz-Picciotto et al., 2006). Microbial dysbiosis and altered metabolic substrates are two examples of environmental features implicated in ASD (MacFabe, 2013). At its core, however, ASD appears to be driven by genetic traits that confer ∼50% heritability, with 90% concordance in monozygotic twins (Tick et al., 2016).

Why has this biologic information failed to yield an accurate biomarker? First, ASD heterogeneity makes it difficult to generalize a single measure to all children. Second, the evolving nature of brain function over the lifespan necessitates that biomarker discovery is performed within a narrow neurodevelopmental window. Third, ever-changing diagnostic criteria create a challenging landscape for patient characterization. Fourth, overlap between ASD and other cognitive/behavioral phenotypes necessitates comparisons with “control” participants exhibiting non-ASD developmental delay (DD). Finally, nearly all studies rely on single molecule types. To date, most ASD biomarker studies fail to overcome these challenges. Few employ sufficient sample sizes, focus on multiple molecule types, or include separate training and test sets, leading to poor generalizability and validity.

Experts have proposed that methods analyzing entire networks of biomarkers may increase the specificity of ASD testing (Walsh et al., 2011; Goldani et al., 2014; Anderson, 2015). Given the array of genetic and environmental risk factors that typify ASD, “poly-omics” approaches that integrate genetic, epigenetic, and metagenomic methods appear well-suited. Indeed, initial studies of both coding (Kong et al., 2012) and non-coding (Mundalil Vasu et al., 2014) transcriptional elements in the peripheral blood of children with ASD have demonstrated predictive potential for identifying ASD. Our pilot study demonstrated that many of these same RNA elements could be detected in saliva of children with ASD (Hicks and Middleton, 2016; Hicks et al., 2016). As a non-invasive approach, collecting saliva boosts sample sizes through increased participation rates, while facilitating interrogation of the microbiome (Frye et al., 2015). Thus, a single high-throughput analysis can interrogate genomic and environmental components implicated in ASD.

Here, we interrogate levels of human and microbial saliva RNAs to train and then test a biomarker classification tool in 456 children, age 19–83 months. This study tests the hypothesis that oral transcriptome measurement provides a broad network perspective that can accurately identify ASD status in children.

## Materials and Methods

### Ethics Statement

This study was carried out in accordance with the recommendations of the Institutional Review Boards (IRBs) at the State University of New York (SUNY) Upstate Medical University, Penn State College of Medicine, and University of California, Irvine (UCI) with written informed consent from all subjects. All subjects gave written informed consent in accordance with the Declaration of Helsinki. The protocol was approved by the SUNY Upstate, Penn State, and UCI IRBs.

### Study Population

The study included 456 children, 19–83 months of age. To our knowledge this is the largest study of RNA expression in children with ASD. Note that the sample is smaller than some DNA-based studies of ASD because these studies rely on measurements of rare-occurring CNVs or SNPs, while the current study focuses on RNA transcripts present in the majority of children (at varying concentrations). Participants were recruited from Penn State (*n* = 250), SUNY Upstate (*n* = 191), and UC Irvine (*n* = 15). 238 children had a clinical diagnosis of ASD, based on DSM-5 criteria. This criterion was chosen to accommodate the phenotypic heterogeneity observed in clinical practice (Anderson, 2015), at an age when initial ASD diagnoses typically occur (Mandell et al., 2005). ASD participants were enrolled following developmental assessment by a trained clinician (e.g., developmental pediatrician or developmental psychologist). ASD participants were compared with 218 control participants: 134 children with typical development (TD) and 84 children with non-ASD DD. TD and DD participants were enrolled following yearly well-child visits or specialist developmental assessment, respectively. For DD participants, absence of ASD was confirmed through negative MCHAT-R and/or clinician assessment with DSM-5 criteria. The majority of DD participants were characterized by expressive speech delay. Exclusion criteria for all groups included feeding tube dependence, active periodontal disease (e.g., unfilled cavities, bottle-rot), active upper respiratory infection, or wards of the state.

General guidelines for interpretation of binomial classification analysis results using receiver operating characteristic (ROC) curves have established that values of 0.5–0.6 reflect nearly worthless classifiers, 0.6–0.7 reflect poor classifiers, 0.7–0.8 reflect fair classifiers, and 0.8–0.9 represent good classifiers. Following these criteria, the goals of our study were to distinguish good classifiers from poor classifiers. Using Power Analysis and Sample Size Software (v15; NCSS, LLC; Kaysville, UT, United States), we thus set the null area under the curve (AUC) upper limit to 0.7, and determined that the sample sizes used in our training set provided 85% power to detect an AUC of the ROC curve = 0.77 (based on a one-sided z-test, with an alpha = 0.05), 99% power to detect an AUC > 0.8, and 100% power to detect an AUC > 0.84. Similarly, the validation cohort (*n* = 84) had 85% power to detect an AUC = 0.85, 90% power to detect an AUC = 0.86, 94.5% power to detect an AUC > 0.87, and 100% power to detect an AUC = 0.90.

### Data Collection

Medical and demographic characterization was performed as follows: age (months), sex, race, gestational age at birth (weeks), and family history of ASD (first- or second-degree relatives) were collected through parent report. Sleep disorder (defined as difficulty initiating sleep, difficulty maintaining sleep, or use of melatonin), gastrointestinal diagnosis (defined as reflux, constipation, chronic diarrhea, or chronic abdominal pain), asthma, and attention deficit hyperactivity disorder (ADHD) were screened through parent report and verified through chart review. Body mass index (BMI; kg/m^2^) was measured at the time of sample collection, or obtained through chart review. Adaptive behavior was assessed with Vineland Adaptive Behavior Scales, 2nd edition (VABS-2) on most (77%) children (*n* = 349). For 128 ASD and 38 DD participants, assessment of autistic traits was performed with the Autism Diagnostic Observation Schedule, 2nd edition (ADOS-2) by trained research staff or obtained from medical records if performed in the past year.

### RNA Collection, Processing, and Quantification

At the time of enrollment, saliva was obtained in a non-fasting state with an Oracollect RNA swab (DNA Genotek; Ottawa, Canada) following water rinse. Pooled saliva was collected by applying the highly absorbent swab at two sites: (1) the base of the tongue (near the sublingual ducts); and (2) bilaterally between the gums and buccal mucosa (proximal to the parotid ducts). Saliva collection was completed in 5–10 s. Scraping of the buccal mucosa and teeth was generally avoided. RNA was extracted from whole saliva with a standard Trizol method (Hicks and Middleton, 2016). Whole saliva was employed rather than exclusive isolation of epithelial or exosomal RNA because each fraction provided complementary information about host and microbial transcription (Park et al., 2006; Michael et al., 2010). For example, exosomal RNA may arise from cranial nerve signaling (which could be disrupted in cases of speech apraxia, or food texture sensitivity), while epithelial RNA may reveal information about transcriptional control within cells. RNA was sequenced at the SUNY Molecular Analysis Core using an Illumina TruSeq Small RNA Prep protocol and a NextSeq500 instrument (Illumina; San Diego, CA, United States) at a targeted depth of ten million, 50 base, single-end reads per sample. Human RNA reads were aligned to the hg38 build of the human genome using Partek Flow (Partek; St. Louis, MO, United States) and the SHRiMP2 aligner. Quantification of aligned RNA reads was performed based on RefSeq annotation, miRbase 21 mature and precursor annotation, and piRNAbase annotation. Microbial transcripts were mapped to the NCBI RefSeq genome database using k-SLAM (Ainsworth et al., 2017). Microbial transcripts were employed in favor of a 16S approach to simplify and streamline nucleic acid extraction and analysis, and facilitate downstream clinical application. In addition, previous studies have demonstrated the potential for microbial RNA to differentiate children with ASD (Hicks et al., 2018).

Samples were randomly divided into a training set (*n* = 372, 82% of samples; 188 ASD, 113 TD, 71 DD samples) in which RNA levels were directly examined, and a test set (*n* = 84, 18% of samples; 50 ASD, 21 TD, 13 DD samples) in which RNA levels were not inspected (beyond read quality) until model features and predictive performance were ascertained. Diagnosis composition of the test and training set were comparable.

For each sample, five subtypes of RNA were quantified: (1) mature/precursor microRNA (miRNA); (2) piwi-interacting RNA (piRNA); (3) non-coding RNA, including small nucleolar RNA (snoRNA) and long intergenic non-coding RNA (lincRNA); (4) ribosomal RNA (rRNA); and (5) microbial RNA. Three samples did not meet quality criteria for inclusion (Hicks et al., 2016) and are not included in the 456 samples. Of the 19,128 RNA features interrogated, 1,078 RNA features contained 99% of the counts per category and were further investigated.

### Data Normalization and Scaling

RNA abundance levels utilized in this study were subjected to a systematic series of data transformations to improve sensitivity for classifier detection and reduce the influence of batch effects. Transformation steps and parameters were determined in the training dataset, and later applied in identical fashion to the hold-out test set (Supplementary Figure S1).

In order to develop robust multivariate classifier models that could utilize RNAs in an unbiased manner across the full expression range, we first employed an inverse hyperbolic sine transformation of the read count data within each RNA category, according to the formula *f*(*x*) = ln$\left(x+\sqrt{x^{2}+1}\right)$ (Burbidge et al., 1988; MacKinnon and Magee, 1990). The rationale for this was based on the common observation that RNA-seq data generally follow non-normal distributions, with some RNAs often expressed at very high levels and others at close to zero (Dimov et al., 2014).

Next, we employed global normalization in which the vector of RNA abundance (within each category) was divided by the norm of the vector (Serneels et al., 2006). This method also imparted robustness to outliers. Together, these steps brought the abundance data within a narrow range, while maintaining relative rank, and served to enhance the likelihood of robust and stable machine learning performance.

To account for subject variability and demographic influences on the classifiers, continuous variables (age, birth age, and BMI) were also subjected to spatial sign transformation to ensure they were commensurate with other variables. Co-morbid medical conditions, history, and race were set to binary factors of 1 (positive/present) or 0 (negative/absent) and reduced to principal components that accounted for 80% of variance.

### Machine Learning Approach

To select and rank the most predictive RNAs within each category, generalized stochastic gradient-boosted logistic models were fit to the training set data. In this method, multivariate logistic models were first trained in an iterative process on subsets of RNAs from subsets of training samples, and input features were given relative ranking based on their prevalence in the logistic models (Friedman, 2001).

Second, to create a joint ranking of all features, the top ranked RNAs from each category were merged with the transformed demographic data and fit to a joint stochastic gradient-boosted model in the training set, as above. This combined model similarly ranked the input features in order of importance across all categories (RNA, biological, demographic, etc.).

Third, to build a predictive model based on these ranked features, radial kernel support vector machines (SVMs) (Cortes and Vapnik, 1995; Chang and Lin, 2001) were trained on increasing numbers of features until model performance on the training set became asymptotic. Outputs from SVMs were converted to probabilities using Platt calibration (Platt, 1999). To reduce overfitting by including too many input features, the SVM with the fewest features that reached the predictive performance plateau was picked as the final model.

As an additional step to help prevent overfitting the training data, 10-fold cross-validation was performed 10 times in each step. Additionally, model parameters (including gradient step size, minimum number of samples per iteration, maximum number of features per logistic model, size of the radial basis function, and cost budget) were carefully selected from reasonable ranges. Confidence intervals for ROC curve performance were determined with the Clopper and Pearson (1934) method following 10-fold cross-validations.

Transformation parameters and loadings calculated on the training set (*n* = 372) were subsequently applied to the completely naïve holdout test set (*n* = 84) to determine ASD-status based on poly-omic RNA concentrations. Misclassification analysis across training and test sets compared the means of various patient features between correctly- and incorrectly predicted participants using analysis of variance (ANOVA).

Data transformation, machine learning implementation, and statistical analyses were performed in R ^1^ using RStudio ^2^, the caret package (Kuhn, 2008), and custom scripts on a HIPAA compliant AWS server ^3^.

### Functional Analysis

Genomic loci for the predictive RNAs were determined using the University of California Santa Cruz Genome Browser^4^. RNA loci were cross-referenced against 2,223 CNV regions associated with ASD on the SFARI database^5^. For predictive miRNAs, high-confidence mRNA targets (microT-CDS scsore ≥ 0.95) were determined in DIANA miRPath v3 software^6^ (Vlachos et al., 2012) and over-represented Kyoto Encyclopedia of Genes and Genomes (KEGG) pathways (FDR < 0.05) were reported. Putative mRNA targets were also cross-referenced against 990 ASD candidate genes from the SFARI database known to contain single nucleotide polymorphisms (SNPs) associated with ASD. Previous reports of candidate miRNAs in human ASD studies were interrogated through published literature review (Hicks and Middleton, 2016). Associations between microbial RNA and human RNA abundance were determined with Pearson Correlation Analysis and hierarchical clustering with complete linkages.

## Results

### Participant Characteristics

The analysis included 456 children in the training and test datasets (mean age 51 ± 16 months; 77% male; 66% Caucasian; 52% with ASD; Table 1). In the training set, the ASD group (*n* = 188) was older, included more males and fewer Caucasians, and had higher rates of disordered sleep, ADHD, and gastrointestinal disturbance than the non-ASD group (*n* = 184). There were no differences between the ASD and non-ASD groups in BMI, asthma rates, or gestational age. In the naïve test set, the ASD group had higher rates of ADHD but did not differ in other medical/demographic factors.

**Table 1:** Participant characteristics.

Clinical characteristics	All (*n* = 456)	Train set (*n* = 372)		Test set (*n* = 84)	
		ASD (188)	Non-ASD (184)	ASD (50)	Non-ASD (34)
**Demographic**					
Male sex, # (%)	337 (76)	156 (83)^∗^	122 (66)	45 (90)	26 (76)
Mean age, mos (SD)	51 (16)	54 (15)^∗^	49 (16)	53 (15)	46 (16)
White race, # (%)	296 (67)	122 (65)^∗^	126 (69)	29 (58)	23 (68)
**Medical**					
BMI, kg/m^2^ (SD)	16.7 (2.5)	16.6 (2.9)	16.8 (2.3)	16.6 (2.1)	16.7 (2.4)
Sleep disorder, # (%)	141 (32)	85 (45)^∗^	31 (17)	21 (42)	8 (24)
ADHD, # (%)	63 (14)	41 (22)^∗^	18 (10)	4 (8)^∗^	0 (0)
GI diagnosis, # (%)	57 (13)	36 (19)^∗^	16 (9)	7 (14)	1 (3)
Asthma, # (%)	43 (10)	16 (9)	18 (10)	6 (12)	2 (6)
Gestation, wks (SD)	38.6 (2.6)	39 (3)	39 (3)	38 (2)	39 (2)
fam hx, # (%)	172 (39)	93 (50)^∗^	51 (28)	29 (58)	10 (29)
**Behavioral**					
VABS Comm (SD)	82.7 (22.8)	72.2 (20.1)^∗^	93.5 (20.7)	73.5 (20.8)^∗^	93.4 (18.2)
VABS Social (SD)	84.7 (22.7)	72.2 (16.4)^∗^	97.4 (22.6)	73.5 (18.0)^∗^	96.3 (15.0)
VABS Adaptive (SD)	84.8 (20.0)	74.9 (15.2)^∗^	95.0 (20.0)	73.6 (18.9)^∗^	96.6 (11.8)
ADOS, mean (SD)	6.1 (2.6)	6.7 (2.4)^∗^	4.5 (2.9)	6.7 (1.6)^∗^	3.2 (1.1)

*Participant characteristics for children in the training and testing datasets. Presence of disordered sleep was ascertained through parental endorsement of difficulty with sleep initiation, sleep maintenance, or use of melatonin. Gastrointestinal (GI) diagnosis denotes documentation of an ICD-10 code related to reflux, constipation, chronic diarrhea, or chronic abdominal pain. Family history (fam hx) of psychological disorders is based on parental report in a first degree relative. For vineland adaptive behavior scales (VABS), standard scores are reported (n = 349). For Autism Diagnostic Observation Schedule (ADOS) evaluation, mean comparison scores are reported (n = 166; ASD and DD groups only). All p-values are based on a two-tailed Student’s t-test, where ^∗^ denotes p < 0.05 for ASD vs. Non-ASD. ^#^Indicates the number of participants with a given clinical characteristic. ASD, autism spectrum disorder; ADHD, attention deficit hyperactivity disorder; BMI, body mass index; mos, months; wks, weeks; Comm, Communication.*

In training and test sets the ASD group displayed lower mean VABS-2 standard scores (*p* < 0.001) in Communication, Socialization, and Adaptive Behavior domains compared with the non-ASD group. For the subset of children evaluated with ADOS-2 (*n* = 166), ASD participants received higher (*p* < 0.001) mean Comparison Scores than DD participants (Table 1).

### RNA Selection and Performance

**FIGURE 1:**
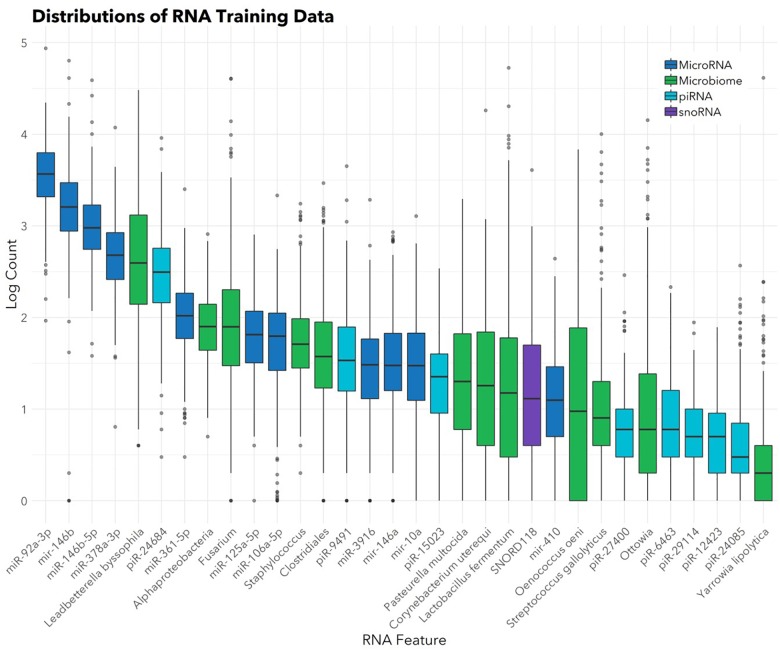
Abundances of 32 salivary RNAs selected for the diagnostic panel. Box and whisker plots show distributions (in the training set; *n* = 372) of the 32 RNAs included in the panel. Color indicates RNA category. Box hinges indicate 25th, 50th, and 75th percentiles. Upper whiskers extend to the largest value up to 1.5^∗^IQR, where IQR is inter-quartile range between the 75th and 25th percentiles. Lower whiskers extend to the smallest value down to 1.5^∗^IQR. Outliers beyond the whiskers are plotted individually.

**FIGURE 2:**
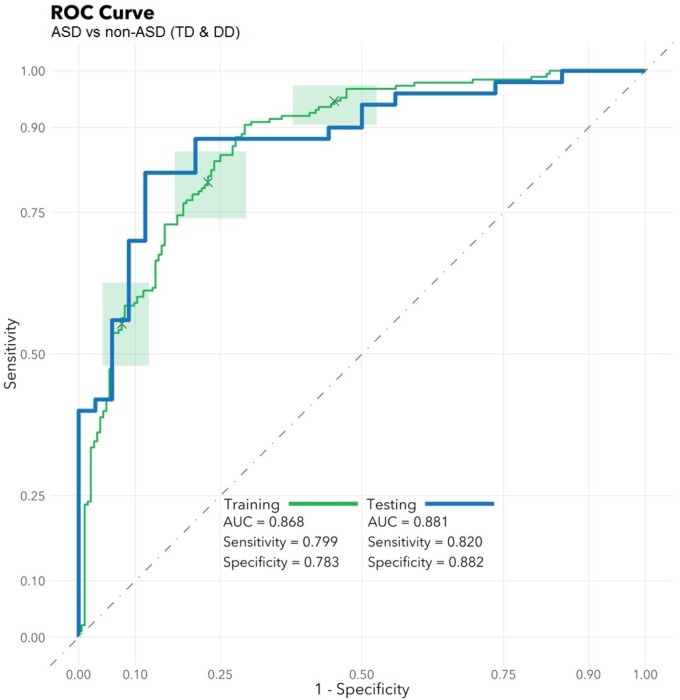
Algorithm Performance. In a training set (*n* = 372), a radial kernel support vector machine model used 32 salivary RNA features (comprising 11 miRNAs, 8 piRNAs, 1 snoRNA, and 12 microbial taxons) to differentiate 188 children with autism spectrum disorder (ASD) from 184 non-ASD peers with typical development (TD) or non-ASD developmental delay (DD), while controlling for participant medical and demographic features. Performance is displayed on the green receiver operator characteristic curve (area under the curve: 0.87; 95% CI: 0.86–0.88) as a function of varying the threshold of probability of ASD prediction. Green boxes indicate rectangular confidence regions with cross-validation. In a naïve test set (*n* = 84), the algorithm correctly identified 41/50 ASD, 18/21 TD, and 12/13 DD children [area under the curve (AUC): 0.88, blue/bold].

The feature selection algorithm resulted in a panel comprised of 32 diagnostic RNA features, including 12 microbial taxa, 7 mature miRNAs, 4 precursor miRNAs, 8 piRNAs, and 1 snoRNA (Figure 1). No rRNAs or lincRNAs were selected. In training set cross-validation, the algorithm identified ASD status with 80% sensitivity and 78% specificity and an AUC of 0.87 (95% CI: 0.86–0.88; Figure 2). When applied to the untrained, naïve test set, the algorithm accurately predicted ASD status in 41/50 ASD children, 18/21 TD children, and 12/13 DD children. This represented an AUC of 0.88, with 82% sensitivity, 88% specificity, and a positive predictive value of 91%.

**FIGURE 3:**
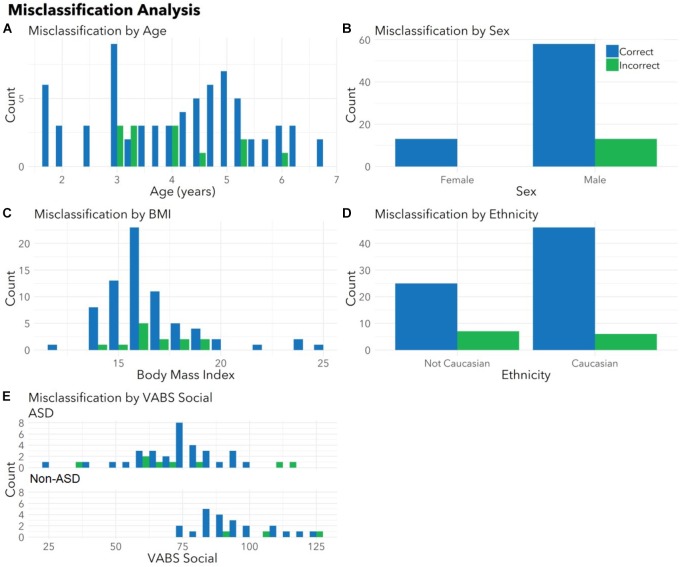
Correctly and incorrectly classified children display similar medical and demographic features. Histograms display distributions of **(A)** age, **(B)** sex, **(C)** body mass index (BMI), **(D)** ethnicity, and **(E)** VABS social for children correctly (blue) and incorrectly (green) classified by autism status in the validation set. There were no differences between correctly and incorrectly classified children in age (*p* = 0.67), sex (*p* = 0.10), race (all *p* > 0.05), BMI (*p* = 0.97), or VABS social (ASD *p* = 0.93, non-ASD *p* = 0.34) for the test set. Histograms combine totals of correctly and incorrectly classified participants from both sets. Note that parents were able to indicate multiple races and the model had access to all races indicated. For this plot, children included as “Caucasian” may include bi-racial participants.

To ensure the classifier algorithm performance was not systematically biased based on patient characteristics, distributions of misclassification errors across patient features were explored in the test set (Figure 3). There were no differences between correctly and incorrectly classified children in age (*p* = 0.67), sex (*p* = 0.10), race (all *p* > 0.05), or BMI (*p* = 0.97). There were no differences between correctly and incorrectly classified children in VABS social score (ASD *p* = 0.92; non-ASD *p* = 0.34). Notably 1/4 of misclassified children with ASD demonstrated above average social scores (>100). Misclassification errors relative to co-morbid medical conditions (Supplementary Table S1) revealed no discernable pattern of confounding influence.

### Functional Interrogation

Of the 20 human RNAs in the panel, 19 (95%) originated from loci with at least one ASD-associated CNV (Supplementary Table S2). The 11 miRNAs had 1,862 high-confidence (microT-CDS score ≥ 0.95) gene targets, and 198 (11%) of these were among the 909 ASD candidate genes in the SFARI database (Supplementary Table S3). miR-106a-5p targeted the largest number of ASD candidate genes (51, 12% of total targets). Six miRNAs (6/11, 55%) were previously reported in human ASD studies (Hicks and Middleton, 2016), and each were identified in multiple tissues.

Evaluation of putative miRNA targets revealed significant enrichment (FDR < 0.05) in 12 KEGG pathways (Supplementary Table S4). Among the pathways neurobiologically relevant to ASD were axon guidance (FDR < 0.001; 26 genes, 8 miRNAs); neurotrophin signaling (FDR = 0.028; 21 genes, 9 miRNAs), and circadian entrainment (FDR = 0.039, 17 genes, 9 miRNAs). Gene targets for the 11 miRNAs also displayed 6 significant (FDR < 0.05) pathway interactions (Supplementary Table S5). Notably, enrichment occurred in metabolism of xenobiotics (FDR = < 0.001, 3 genes, 2 miRNAs) and NF-kappa B signaling (FDR < 0.001, 2 genes, 2 miRNAs).

Hierarchical clustering analysis of the classifier features revealed two distinct RNA clusters (Supplementary Figure S2). One cluster included human RNA only. A second cluster included eight microbes, two human miRNAs (miR-410, miR-3916), and two human piRNAs (piR-6463, piR-29114). The relationship between these features was further explored using correlation analysis, which revealed moderate associations between piR-6463 and Clostridiales (*R* = 0.46, *p* < 0.001), *Pasteurella multocida* (*R* = 0.46, *P* < 0.001), and *Leadbetterella byssophilia* (*R* = 0.42, *p* < 0.001). piR-29114 also displayed associations with Clostridiales (*R* = 0.42, *p* < 0.001).

## Discussion

This investigation identified 32 salivary RNA features that accurately distinguished ASD status in a training set of 372 children, and displayed 85% accuracy in a separate test set of 84 additional children. The RNA panel included human RNAs and microbial RNAs with putative functions converging on ASD-associated neurobiological pathways. It provides an accurate, objective, systems-based method for identifying ASD status.

### Clinical Implications

The ability to clinically discern children with autistic from peers with non-ASD DD is more challenging than discriminating ASD from TD children. Yet, children with DD have typically not been included in ASD biomarker studies. In this study, the RNA algorithm identified 41/50 ASD children while differentiating 30/34 non-ASD children in a naïve hold-out sample set. Notably, test performance was similar for TD (18/21) and DD (12/13) children. The potential to accurately discriminate between ASD and DD lies at the crux of ASD diagnoses, and represents a significant potential contribution for this objective, biologic assay.

This study employed ASD and non-ASD groups of equal size, and the total non-ASD cohort included 39% DD participants (84/218). Thus, this algorithm is not designed as a screening tool (where ∼1/50 children might have ASD). Instead, our results are best viewed as an adjunct to positive MCHAT-R screening, or as an aid in ASD diagnosis. In these settings (e.g., after a positive MCHAT-R), nearly 50% of children would be expected to have ASD, and a significant proportion of the others would likely have DD.

Although our test differentiates ASD and non-ASD participants from multiple geographic regions, whether the algorithm performs accurately in populations with increasing geographic diversity remains to be determined. This is particularly important to consider given that our algorithm includes microbial RNAs, which could be influenced by dietary and environmental factors. The present algorithm was developed from saliva of children residing in New York and Pennsylvania. When applied to a test set that contained children from New York, Pennsylvania, and California, the test maintained diagnostic accuracy. Future investigations will need to validate these findings across broader geographic cohorts. Refinement of the model may improve performance further as we continue to sample increasingly diverse populations at high volumes and incorporate data into the training steps.

The potential to employ this test in younger toddlers and infants has yet to be assessed. While it is possible that the modest difference in age between the ASD and non-ASD groups may have confounded the analyses, the children incorrectly classified displayed similar age, sex, and race as those who were correctly classified. Thus, the algorithm showed no bias toward demographic or medical factors within the study cohort. It also suggests the test may be broadly applicable without exclusion of medical or demographic subgroups. Conditions that might impact salivary RNA (e.g., asthma, gastrointestinal disturbance, BMI) and conditions that are more common in children with ASD (sleep difficulties, ADHD) also did not appear to bias ASD prediction.

This study recruited a cohort generally representative of children receiving developmental referral, favoring robust statistical power over extensive phenotypic analyses. Group assignments were based on clinical assessments. Participant characterization was driven by a combination of parent report, chart review, and standardized VABS-2 and ADOS-2 assessments. Future studies employing extensive behavioral assessment alongside longitudinal RNA sampling and therapeutic interventions may identify nuances in salivary RNA profiles that correlate with ASD endophenotypes, respond to intervention, or prove useful for guiding personalized therapies.

### Physiologic Implications

**FIGURE 4:**
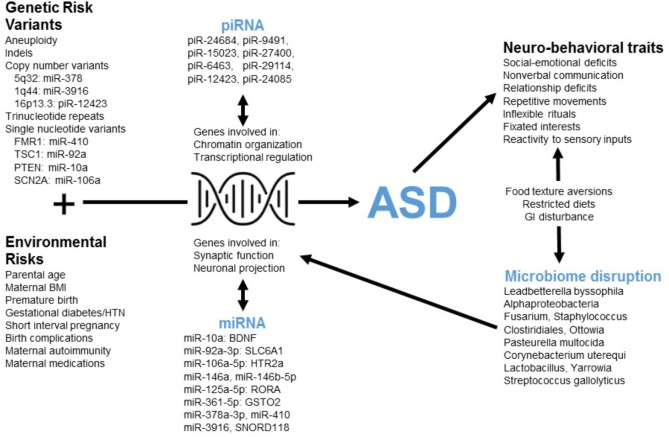
The poly-omic diagnostic panel integrates genetic, neurobiologic, phenotypic, and environmental factors implicated in ASD. This concept diagram displays the putative function of human RNAs at the intersection between genetic/environmental risk factors and the neuro-behavioral traits associated with ASD. Factors, such as microRNA (miRNA) and piwi-interacting RNA (piRNA) interact with genes involved in chromatin organization, transcriptional regulation, synaptic function, and other critical neuronal pathways. Disruption of these pathways in children with ASD may lead to alterations in the levels of peripheral miRNAs and piRNAs. In addition, neuro-behavioral characteristics of ASD, which can lead to restricted diets and gastrointestinal (GI) disturbance, may be related to the microbial disruptions upon which the current algorithm is based.

This tool employs poly-omic RNA measures that link both physiologic and environmental factors implicated in ASD (Figure 4). The transcript dysregulation that is apparent in ASD children may arise in response to genetic alterations and cause down-stream changes in neurobiological pathways. Such changes could form the basis for ASD behavior, and some of these behaviors (e.g., restricted diet, difficulty with dental hygiene) may lead to dysbiosis. Thus, transcriptional measurements provide a broad network perspective with the potential to unify the heterogeneity of ASD.

Three RNAs in the current panel (miR-378a, miR-3916, piR-12423) arise from loci associated with ASD copy number variants (CNVs) (Figure 4). The results do not imply that these genomic regions are commonly altered in children with ASD (and the lack of genomic sequencing in our cohort prevents identification of common CNVs or syndromic cases). Instead, variations in non-coding RNA abundance may arise in response to dysregulation of several neurobiologic systems that have been previously reported in ASD literature. For instance, several miRNAs in the panel target mRNAs from ASD-candidate genes, including miR-410/FMR1, miR-10a/PTEN, miR-92a/TSC1, and miR-106a/SCN2A. An additional member of the RNA panel, SNORD118, may play a critical role in ribosomal function and protein translation (Jenkinson et al., 2016): a study of 40 patients having leukoencephalopathy with calcifications and cysts identified bi-allelic mutations in SNORD118.

The miRNAs in this panel target transcripts that code for critical elements of neurotransmitter (miR-10a/BDNF; miR-92a/GABA), neurohormonal (miR-106a/serotonin), immune (miR-106a/TGF-beta; miR-106a/TNF-alpha) and xenobiotic (miR-361/GSTO2; miR-125a/GSTM2) pathways (Figure 4). Individual miRNA targets also demonstrate pathway interactions for critical immune components (NF Kappa B and Toll-like receptor signaling; Supplementary Table S5). Given this immune enrichment, host transcripts may be directly linked to ASD dysbiosis. This is supported by the finding that four human RNAs and eight microbes cluster based on salivary abundance (Supplementary Figure S2). Some microbes in the panel may contribute to ASD neurobiology through effects on host metabolism [*Oenococcus oeni*/pyruvate (Wagner et al., 2005); *Pasteurella multocida*/ammonia (Hamilton et al., 1996)] and gastrointestinal inflammation [Clostridiales (Frank et al., 2007)]. Alternatively, alterations in oral microbe transcription may result from host dietary restrictions [e.g., Lactobacillus (Mikelsaar and Zilmer, 2009)].

### Limitations

Reliance on microbial measures for ASD identification will require accounting for features influenced by diet and geography. The current study enrolled children from multiple sites and relied on several microbes found in humans throughout the world (e.g., Lactobacillus). However, validation of RNA from less common bacteria (e.g., *Oenococcus oeni*) will require sample collection from diverse sites. In our prior study of the salivary micro-transcriptome, the majority of RNA transcripts “altered” in children with ASD showed no relationship with dietary restrictions (Hicks et al., 2018). Thus, we expect that geographic variations in diet will have minimal impact on most microbial biomarkers.

Numerous medical and demographic factors may influence RNA expression in the oropharynx. We have attempted to control for these factors (e.g., BMI, asthma) through matched recruitment and a statistical modeling approach controlling for medical/demographic factors. Inherent differences in rates of gastrointestinal disturbance between ASD and non-ASD groups (Serneels et al., 2006) likely drive some transcriptional changes that contribute to test performance. Because medical and demographic features of our cohort generally represent childhood ASD populations, we expect these differences will not impact external validity. Indeed, in the test set (which was matched on ASD:TD:DD ratios, but not medical and demographic factors) the RNA panel maintains predictive accuracy.

## Conclusion

We have developed an objective, quantitative algorithm based on salivary RNA abundance that accurately discriminates children with ASD from peers with DD or TD. This non-invasive test could augment the accuracy of current ASD assessment, as an adjunctive tool for children with positive MCHAT screening, or an objective aid in ASD diagnosis.

## Data Availability

The datasets used and/or analyzed during the current study are not available to the public.

## Author Contributions

SH and FM conceived the study. SH, FM, and RC contributed to study design. KW and SB coordinated the participant enrollment and data collection. SH, AR, and FM carried out data analysis. SH and AR contributed equally to the initial draft of the manuscript which was critically reviewed by all authors prior to submission.

## Conflict of Interest Statement

SH, FM, and AR are co-inventors of patent applications for RNA biomarkers in autism spectrum disorder that is assigned to The Research Foundation for the State University of New York, The Penn State Research Foundation and Quadrant Biosciences Inc., and licensed to Quadrant Biosciences Inc. FM and SH serve on the scientific and medical advisory boards of Quadrant Biosciences Inc., and SH is a paid consultant for Quadrant Biosciences Inc. These conflicts of interest are actively managed by the Penn State College of Medicine. AR, RC, KW, and SB are employees of Quadrant Biosciences Inc.

## Footnotes

1 r-project.org

2 rstudio.com

3 aws.amazon.com

4 https://genome.ucsc.edu/

5 https://gene.sfari.org/database/cnv/

6 http://diana.imis.athena-innovation.gr/DianaTools/index.php

## Supplementary Material

FIGURE S1 Algorithm training and testing. The methodological pipeline used for RNA feature selection and model development in the training set is shown, along with direct application of the diagnostic algorithm to the naïve hold out test samples.

FIGURE S2 Associations between human and microbial salivary RNA features. Hierarchical clustering of the 32 RNA classifiers was performed using Pearson Correlation Analysis with complete linkages. There were two distinct RNA clusters: one involving solely human RNA, and a second containing 8 microbes alongside 2 microRNAs and 2 piRNAs. This second cluster may denote human and microbe elements with functional interactions.

TABLE S1 Rates of medical conditions in correctly and incorrectly classified participants. The proportion of correctly and incorrectly classified children in the naïve validation set (*n* = 84) with various co-morbid medical conditions are shown. Overall, no medical condition was commonly present only among correctly or incorrectly classified groups, suggesting that the algorithm was not biased by these conditions.

TABLE S2 Human RNA loci with autism-associated copy number variants. The genomic location for each of the human RNA classifiers is shown, along with the number of autism-associated CNVs for this region, autism-related case reports, autism cases in the Simons Population, and total reported human cases in the Simons Foundation database.

TABLE S3 ASD Candidate Genes Targeted by the 11 microRNAs. All autism-associated genes (Simons Foundation database) targeted with high confidence (based on microT-CDS score) by the 11 microRNA classifiers are listed. The strength of the microRNA-gene interaction is listed (Target Score), and if the interaction has been experimentally validated it is noted. Simons Foundation characteristics for each gene are noted, including gene score (strength of autism-association), implication in syndromic forms of autism, and total number of autistic individuals with a known variant in the gene.

TABLE S4 KEGG Pathways over-represented by microRNA targets. The KEGG pathways over-represented (FDR < 0.05) by high-confidence gene targets of the microRNA classifiers are listed. The number of genes targeted within each pathway are shown along with the number of microRNAs targeting them. Note that several brain-related pathways have enriched numbers of targets, including axon guidance, neurotrophin signaling, and glioma.

TABLE S5 Pathway interactions among putative microRNA targets. DIANA miRPATH software was used to identify KEGG pathways whose gene components had putative interacting relationships with the 11 microRNA classifiers. Notably, several pathways involved in metabolism and immune pathways previously implicated in autism were on the list.

## Abbreviations

ADHD: attention deficit hyperactivity disorder
ADOS: Autism Diagnostic Observation Schedule
ANOVA: analysis of variance
ASD: autism spectrum disorder
AUC: area under the curve
BDNF: brain-derived neurotrophic factor
BMI: body mass index
CNV: copy number variant
DD: developmental delay
DSM: diagnostics and statistics manual
GABA: gamma-amino butyric acid
linc RNA: long intergenic non-coding RNA
MCHAT-R: modified checklist for autism in toddlers – revised
miRNA: microRNA
piRNA: piw-ineracting RNA
ROC: receiver operating characteristics
rRNA: ribosomal RNA
SFARI: simons foundation autism research initiative
snoRNA: small nucleolar RNA
SNP: single nucleotide polymorphism
SUNY: State University of New York
SVM: support vector machine
TD: typical development
UCI: University of California Irvine
VABS: Vineland Adaptive Behavior Scales.

## References

[B1] Ainsworth D. Sternberg M. J. E. Raczy C. Butcher S. A. (2017). k-SLAM: accurate and ultra-fast taxonomic classification and gene identification for large metagenomic data sets. *Nucleic Acids Res.* 45 1649–1656. 10.1093/nar/gkw1248 27965413 PMC5389551

[B2] Anderson G. M. (2015). Autism biomarkers: challenges, pitfalls and possibilities. *J. Autism Dev. Disord.* 45 1103–1113. 10.1007/s10803-014-2225-4 25193140

[B3] Bisgaier J. Levinson D. Cutts D. B. Rhodes K. V. (2011). Access to autism evaluation appointments with developmental-behavioral and neurodevelopmental subspecialists. *Arch. Pediatr. Adolesc. Med.* 165 673–674. 10.1001/archpediatrics.2011.90 21727283

[B4] Burbidge J. B. Magee L. Robb A. L. (1988). Alternative transformations to handle extreme values of the dependent variable. *J. Am. Statist. Assoc.* 83 123–127. 10.1080/01621459.1988.10478575

[B5] Chamberlain R. S. Herman B. H. (1990). A novel biochemical model linking dysfunctions in brain melatonin, proopiomelanocortin peptides, and serotonin in autism. *Biol. Psychiatry* 28 773–793. 10.1016/0006-3223(90)90513-2 2175218

[B6] Chang C. C. Lin C. J. (2001). LIBSVM: a library for support vector machines. *ACM Trans. Intell. Syst. Technol.* 2:27.

[B7] Chauhan A. Chauhan V. (2006). Oxidative stress in autism. *Pathophysiology* 13 171–181. 10.1016/j.pathophys.2006.05.007 16766163

[B8] Clopper C. J. Pearson E. S. (1934). The use of confidence or fiducial limits illustrated in the case of the binomial. *Biometrika* 26 404–413. 10.1093/biomet/26.4.404

[B9] Connolly A. M. Chez M. Streif E. M. Keeling R. M. Golumbek P. T. Kwon J. M. (2006). Brain-Derived neurotrophic factor and autoantibodies to neural antigens in sera of children with autistic spectrum disorders, landau-kleffner syndrome, and epilepsy. *Biol. Psychiatry* 59 354–363. 10.1016/j.biopsych.2005.07.004 16181614

[B10] Corbett B. A. Kantor A. B. Schulman H. Walker W. L. Lit L. Ashwood P. (2006). A proteomic study of serum from children with autism showing differential expression of apolipoproteins and complement proteins. *Mol. Psychiatry* 12:292. 17189958 10.1038/sj.mp.4001943

[B11] Cortes C. Vapnik V. (1995). Support-vector network. *Mach. Learn.* 20 1–25. 10.1007/BF00994018

[B12] Dimov I. K. Lu R. Lee E. P. Seita J. Sahoo D. Park S. M. (2014). Discriminating cellular heterogeneity using microwell-based RNA cytometry. *Nat. Commun.* 5:3451. 10.1038/ncomms4451 24667995 PMC4075946

[B13] El-Ansary A. Al-Ayadhi L. (2014). GABAergic/glutamatergic imbalance relative to excessive neuroinflammation in autism spectrum disorders. *J. Neuroinflam.* 11:189. 10.1186/s12974-014-0189-0 25407263 PMC4243332

[B14] Frank D. N. St Amand A. L. Feldman R. A. Boedeker E. C. Harpaz N. Pace N. R. (2007). Molecular-phylogenetic characterization of microbial community imbalances in human inflammatory bowel diseases. *Proc. Natl. Acad. Sci. U.S.A.* 104 13780–13785. 10.1073/pnas.0706625104 17699621 PMC1959459

[B15] Friedman J. H. (2001). Greedy function approximation: a gradient boosting machine. *Ann. Statist.* 29 1189–1232. 10.1214/aos/1013203451

[B16] Frye R. E. Rose S. Slattery J. MacFabe D. F. (2015). Gastrointestinal dysfunction in autism spectrum disorder: the role of the mitochondria and the enteric microbiome. *Microb. Ecol. Health Dis.* 26:27458. 10.3402/mehd.v26.27458 25956238 PMC4425813

[B17] Geschwind D. H. (2008). Autism: Many Genes. Common Pathways? *Cell* 135 391–395. 10.1016/j.cell.2008.10.016 18984147 PMC2756410

[B18] Goines P. Van de Water J. (2010). The immune system’s role in the biology of autism. *Curr. Opin. Neurol.* 23 111–117. 10.1097/WCO.0b013e3283373514 20160651 PMC2898160

[B19] Goldani A. A. Downs S. R. Widjaja F. Lawton B. Hendren R. L. (2014). Biomarkers in autism. *Front. Psychiatry* 5:100. 10.3389/fpsyt.2014.00100 25161627 PMC4129499

[B20] Hamilton T. Row J. Webster A. (1996). Synergistic role of gaseous ammonia in etiology of *Pasteurella multocida*-induced atrophic rhinitis in swine. *J. Clin. Microbiol.* 34 2185–2190. 8862582 10.1128/jcm.34.9.2185-2190.1996 PMC229214

[B21] Hertz-Picciotto I. Croen L. A. Hansen R. Jones C. R. van de Water J. Pessah I. N. (2006). The CHARGE study: an epidemiologic investigation of genetic and environmental factors contributing to autism. *Environ. Health Perspect.* 114 1119–1125. 10.1289/ehp.8483 16835068 PMC1513329

[B22] Hicks S. D. Ignacio C. Gentile K. Middleton F. A. (2016). Salivary miRNA profiles identify children with autism spectrum disorder, correlate with adaptive behavior, and implicate ASD candidate genes involved in neurodevelopment. *BMC Pediatr.* 16:52. 10.1186/s12887-016-0586-x 27105825 PMC4841962

[B23] Hicks S. D. Middleton F. A. (2016). A comparative review of microRNA expression patterns in autism spectrum disorder. *Front. Psychiatry* 7:176. 10.3389/fpsyt.2016.00176 27867363 PMC5095455

[B24] Hicks S. D. Uhlig R. Afshari P. Williams J. Chroneos M. Tierney-Aves C. (2018). The unique oral microbiome of autism spectrum disorder. *Autism Res.* 10.1002/aur.1972 [Epub ahead of print]. 30107083 PMC7775619

[B25] Jenkinson E. M. Rodero M. P. Kasher P. R. Uggenti C. Oojageer A. Goosey L. C. (2016). Mutations in SNORD118 cause the cerebral microangiopathy leukoencephalopathy with calcifications and cysts. *Nat. Genet.* 48 1185–1192. 10.1038/ng.3661 27571260 PMC5045717

[B26] Kong S. W. Collins C. D. Shimizu-Motohashi Y. Holm I. A. Campbell M. G. Lee I. H. (2012). Characteristics and predictive value of blood transcriptome signature in males with autism spectrum disorders. *PLoS One* 7:e49475. 10.1371/journal.pone.0049475 23227143 PMC3515554

[B27] Kuhn M. (2008). Building predictive models in r using the caret package. *J. Statist. Softw.* 26:5.

[B28] MacFabe D. (2013). Autism: metabolism, mitochondria, and the microbiome. *Glob. Adv. Health Med.* 2 52–66. 10.7453/gahmj.2013.089 24416709 PMC3865378

[B29] MacKinnon J. G. Magee L. (1990). Transforming the dependent variable in regression models. *Int. Econ. Rev.* 31 315–339. 10.2307/2526842

[B30] Mandell D. S. Novak M. M. Zubritsky C. D. (2005). Factors associated with age of diagnosis among children with autism spectrum disorders. *Pediatrics* 116 1480–1486. 10.1542/peds.2005-0185 16322174 PMC2861294

[B31] Michael A. Bajracharya S. D. Yuen P. S. Zhou H. Star R. A. Illei G. G. (2010). Exosomes from human saliva as a source of microRNA biomarkers. *Oral Dis.* 16 34–38. 10.1111/j.1601-0825.2009.01604.x 19627513 PMC2844919

[B32] Mikelsaar M. Zilmer M. (2009). Lactobacillus fermentum ME-3 – an antimicrobial and antioxidative probiotic. *Microb. Ecol. Health Dis.* 21 1–27. 10.1080/08910600902815561 19381356 PMC2670518

[B33] Mundalil Vasu M. Anitha A. Thanseem I. Suzuki K. Yamada K. Takahashi T. (2014). Serum microRNA profiles in children with autism. *Mol Autism* 5:40. 10.1186/2040-2392-5-40 25126405 PMC4132421

[B34] Onore C. Careaga M. Ashwood P. (2012). The role of immune dysfunction in the pathophysiology of autism. *Brain Behav. Immun.* 26 383–392. 10.1016/j.bbi.2011.08.007 21906670 PMC3418145

[B35] Park N. J. Li Y. Yu T. Brinkman B. M. Wong D. T. (2006). Characterization of RNA in saliva. *Clin. Chem.* 52 988–994. 10.1373/clinchem.2005.063206 16601067 PMC7108156

[B36] Platt J. (1999). Probabilistic outputs for support vector machines and comparisons to regularized likelihood methods. *Adv. Large Marg. Classif.* 10 61–74.

[B37] Serneels S. De Nolf E. Van Espen P. J. (2006). Spatial sign preprocessing: a simple way to impart moderate robustness to multivariate estimators. *J. Chem. Inf. Model.* 46 1402–1409. 10.1021/ci050498u 16711760

[B38] Siu A. L. (US) Preventive Services Task Force (USPSTF) Bibbins-Domingo K. Grossman D. C. Baumann L. C. Davidson K. W. (2016). Screening for autism spectrum disorder in young children: Us preventive services task force recommendation statement. *JAMA* 315 691–696. 10.1001/jama.2016.0018 26881372

[B39] Tick B. Bolton P. Happé F. Rutter M. Rijsdijk F. (2016). Heritability of autism spectrum disorders: a meta-analysis of twin studies. *J. Child Psychol. Psychiatry* 57 585–595. 10.1111/jcpp.12499 26709141 PMC4996332

[B40] Vlachos I. S. Kostoulas N. Vergoulis T. Georgakilas G. Reczko M. Maragkakis M. (2012). DIANA miRPath v.2.0: investigating the combinatorial effect of microRNAs in pathways. *Nucleic Acids Res.* 40 W498–W504. 10.1093/nar/gks494 22649059 PMC3394305

[B41] Wagner N. Tran Q. H. Richter H. Selzer P. M. Unden G. (2005). Pyruvate fermentation by *Oenococcus oeni* and *Leuconostoc mesenteroides* and role of pyruvate dehydrogenase in anaerobic fermentation. *Appl. Environ. Microbiol.* 71 4966–4971. 10.1128/AEM.71.9.4966-4971.2005 16151074 PMC1214600

[B42] Walsh P. Elsabbagh M. Bolton P. Singh I. (2011). In search of biomarkers for autism: scientific, social and ethical challenges. *Nat. Rev. Neurosci.* 12:603. 10.1038/nrn3113 21931335

