# Supplementary figures and images for: Validation of a Salivary RNA Test for Childhood Autism Spectrum Disorder
Source: Front Genet. 2018 Nov 9;9:534. doi: 10.3389/fgene.2018.00534 (PMC6237842; doi:10.3389/fgene.2018.00534)

# ALGORITHM PIPELINE

## ALGORITHM TRAINING

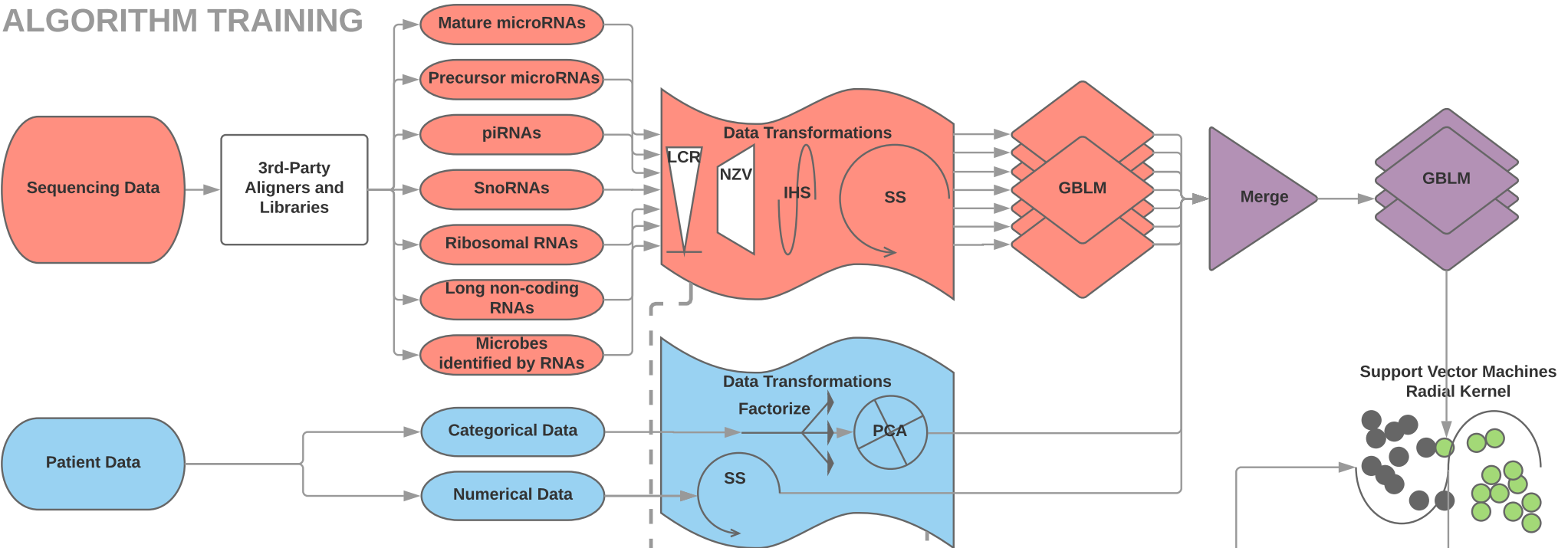

## ALGORITHM TESTING

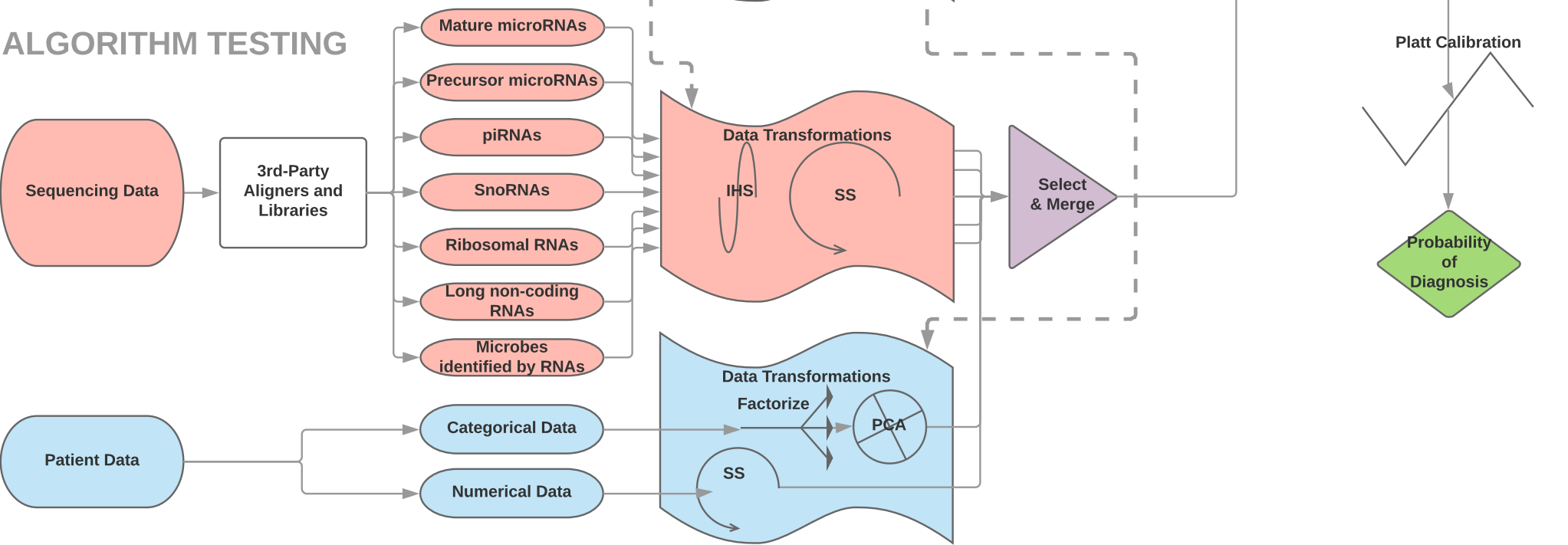

Supplement: FIGURE S1 — Algorithm training and testing. The methodological pipeline used for RNA feature selection and model development in the training set is shown, along with direct application of the diagnostic algorithm to the naïve hold out test samples. [file Image_1.pdf]
