# Supplementary material for: Validation of a Salivary RNA Test for Childhood Autism Spectrum Disorder
Source: Front Genet. 2018 Nov 9;9:534. doi: 10.3389/fgene.2018.00534 (PMC6237842; doi:10.3389/fgene.2018.00534)
Supplement: FIGURE S2 — Associations between human and microbial salivary RNA features. Hierarchical clustering of the 32 RNA classifiers was performed using Pearson Correlation Analysis with complete linkages. There were two distinct RNA clusters: one involving solely human RNA, and a second containing 8 microbes alongside 2 microRNAs and 2 piRNAs. This second cluster may denote human and microbe elements with functional interactions. [file Image_2.pdf]

Additional Figure 2. Associations between human and microbial salivary RNA features

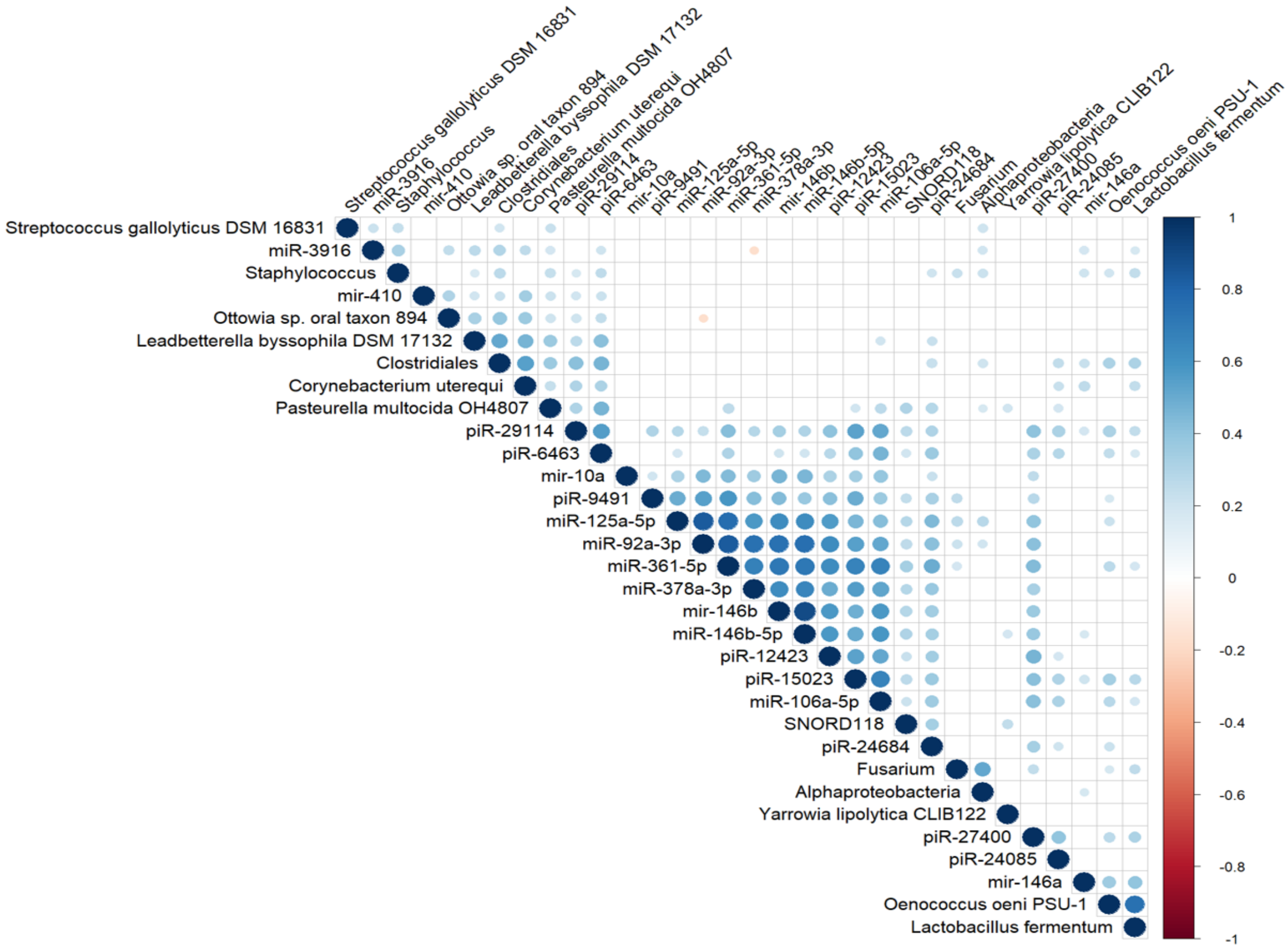

Relationships between microbial and human RNA feature abundance were determined for the 31 selected features with a Pearson correlation analysis. Features were hierarchically clustered based on strength of association. Strength of association is denoted by circle size and direction is noted by color (where blue represents direct and red represents indirect relationships). Only relationships with p<0.01 are shown.
