# Supplementary material for: Validation of a Salivary RNA Test for Childhood Autism Spectrum Disorder
Source: Front Genet. 2018 Nov 9;9:534. doi: 10.3389/fgene.2018.00534 (PMC6237842; doi:10.3389/fgene.2018.00534)
Supplement: TABLE S1 — Rates of medical conditions in correctly and incorrectly classified participants. The proportion of correctly and incorrectly classified children in the naïve validation set (n = 84) with various co-morbid medical conditions are shown. Overall, no medical condition was commonly present only among correctly or incorrectly classified groups, suggesting that the algorithm was not biased by these conditions. [file Table_1.DOCX]

**Additional Table 1. Rates of medical conditions in correctly and incorrectly classified participants**

| **Medical condition** | **Correctly classified (n=71), no. (%)** | **Incorrectly classified (n=13), no. (%)** |
| --- | --- | --- |
| ADHD | 4 (6) | 0 (0) |
| GI disturbance | 8 (11) | 0 (0) |
| Sleep disturbance | 26 (37) | 3 (23) |
| Asthma | 8 (11) | 0 (0) |
| Eczema | 10 (14) | 1 (8) |
| Seasonal allergies | 20 (28) | 1 (8) |
| Daily medication use | 4 (6) | 1 (8) |
| Medical condition | 27 (38) | 1 (8) |

Abbreviations: Attention deficit hyperactivity disorder (ADHD); gastrointestinal (GI).

Parent-reported medical conditions confirmed by chart review. Children with a “medical condition” include those with reported ADHD, GI disturbance, asthma, eczema, seasonal allergies, or “other medical condition” on parental survey.
