# Supplementary material for: Validation of a Salivary RNA Test for Childhood Autism Spectrum Disorder
Source: Front Genet. 2018 Nov 9;9:534. doi: 10.3389/fgene.2018.00534 (PMC6237842; doi:10.3389/fgene.2018.00534)
Supplement: TABLE S2 — Human RNA loci with autism-associated copy number variants. The genomic location for each of the human RNA classifiers is shown, along with the number of autism-associated CNVs for this region, autism-related case reports, autism cases in the Simons Population, and total reported human cases in the Simons Foundation database. [file Table_2.docx]

**Additional Table 2. Human RNA loci and autism-associated CNV regions**

| **RNA Feature** | **Location** | **Overlapping CNV regions** | **Reports (#)** | **Cases-population (#)** | **Cases-individuals (#)** |
| --- | --- | --- | --- | --- | --- |
| miR-106a-5p | Xq26.3 | 4 | 14 | 22 | 83 |
| mir-10a | 17q21.33 | 5 | 10 | 19 | 21 |
| miR-125a-5p | 19q13.41 | 4 | 23 | 42 | 194 |
| mir-146a | 5q34 | 4 | 9 | 19 | 53 |
| mir-146b | 10q24.31 | 2 | 2 | 2 | 2 |
| miR-146b-5p | 10q24.31 | 2 | 2 | 2 | 2 |
| miR-361-5p | Xq21.2 | 2 | 7 | 11 | 12 |
| miR-378a-3p | 5q32 | 2 | 25 | 44 | 370 |
| miR-3916 | 1q44 | 2 | 47 | 72 | 592 |
| miR-410 | 14q32.32 | 2 | 2 | 2 | 2 |
| miR-92a-3p | Xq26.3 | 4 | 14 | 22 | 83 |
| piR-hsa-6463 | Yq11.223 | 6 | 9 | 15 | 19 |
| piR-hsa-15023 | 8q13.2 | 4 | 11 | 20 | 38 |
| piR-hsa-9491 | M 12169-12201 | 0 | 0 | 0 | 0 |
| piR-hsa-27400 | 1q21.3 | 5 | 24 | 38 | 95 |
| piR-hsa-12423 | 16p13.3 | 2 | 45 | 70 | 465 |
| piR-hsa-24085 | 10q11.23 | 4 | 29 | 49 | 75 |
| piR-hsa-24684 | 1q21.2 | 3 | 22 | 33 | 92 |
| piR-hsa-29114 | 17p13.1 | 2 | 2 | 2 | 2 |
| SNORD118 | 17p13.1 | 2 | 2 | 2 | 2 |
