# Supplementary material for: Validation of a Salivary RNA Test for Childhood Autism Spectrum Disorder
Source: Front Genet. 2018 Nov 9;9:534. doi: 10.3389/fgene.2018.00534 (PMC6237842; doi:10.3389/fgene.2018.00534)
Supplement: TABLE S3 — ASD Candidate Genes Targeted by the 11 microRNAs. All autism-associated genes (Simons Foundation database) targeted with high confidence (based on microT-CDS score) by the 11 microRNA classifiers are listed. The strength of the microRNA-gene interaction is listed (Target Score), and if the interaction has been experimentally validated it is noted. Simons Foundation characteristics for each gene are noted, including gene score (strength of autism-association), implication in syndromic forms of autism, and total number of autistic individuals with a known variant in the gene. [file Table_3.docx]

**Additional Table 3. ASD Candidate Genes Targeted by the 11 microRNAs**

| **MicroRNA** | **Gene** | **Target Score** | **Experiment Supported** | **SFARI**  **Gene score** | **Syndromic** | **Reports (#)** | |
| --- | --- | --- | --- | --- | --- | --- | --- |
| miR-92a-3p | TSC1 | 0.97 | No | 0 | 0 | 8 | |
| miR-92a-3p | BRAF | 0.995 | Yes | 0 | 0 | 2 | |
| miR-92a-3p | RBM27 | 0.981 | Yes | 0 | 0 | 1 | |
| miR-92a-3p | RNF38 | 0.999 | Yes | 0 | 0 | 1 | |
| miR-92a-3p | CSMD1 | 0.972 | No | 0 | 0 | 0 | |
| miR-92a-3p | SIK1 | 0.998 | Yes | 0 | 0 | 0 | |
| miR-92a-3p | ZNF804A | 0.986 | No | 0 | 0 | 0 | |
| miR-92a-3p | ADCY3 | 0.999 | No | 0 | 0 | 0 | |
| miR-92a-3p | ROBO2 | 0.997 | No | 0 | 0 | 0 | |
| miR-92a-3p | PCDH11X | 1 | No | 0 | 0 | 0 | |
| miR-92a-3p | ARID1B | 0.986 | Yes | 0 | 0 | 0 | |
| miR-92a-3p | BCL11A | 0.999 | Yes | 0 | 0 | 0 | |
| miR-92a-3p | SYNJ1 | 0.973 | Yes | 0 | 0 | 0 | |
| miR-92a-3p | FMR1 | 0.999 | No | 0 | 0 | 0 | |
| miR-92a-3p | DLGAP2 | 0.991 | No | 0 | 0 | 0 | |
| miR-92a-3p | DDX3X | 0.987 | Yes | 0 | 0 | 0 | |
| miR-92a-3p | USP45 | 0.952 | Yes | 0 | 0 | 0 | |
| miR-92a-3p | CACNA1I | 0.994 | No | 0 | 0 | 0 | |
| miR-92a-3p | CAMK2A | 1 | No | 0 | 0 | 0 | |
| miR-92a-3p | EFR3A | 0.994 | Yes | 0 | 0 | 0 | |
| miR-92a-3p | CTTNBP2 | 0.988 | No | 0 | 0 | 0 | |
| miR-92a-3p | ATRX | 0.991 | Yes | 0 | 0 | 0 | |
| miR-92a-3p | ITPR1 | 0.961 | Yes | 0 | 0 | 0 | |
| miR-92a-3p | RORA | 0.991 | Yes | 0 | 0 | 0 | |
| miR-92a-3p | CUX1 | 0.996 | Yes | 0 | 0 | 0 | |
| miR-92a-3p | WDFY3 | 0.976 | No | 0 | 0 | 0 | |
| miR-92a-3p | MARK1 | 0.963 | No | 0 | 0 | 0 | |
| miR-92a-3p | KAT2B | 0.999 | Yes | 0 | 0 | 0 | |
| miR-92a-3p | LRRC1 | 0.963 | No | 0 | 0 | 0 | |
| miR-92a-3p | NFIA | 0.994 | Yes | 0 | 0 | 0 | |
| miR-92a-3p | PER2 | 0.965 | Yes | 0 | 0 | 0 | |
| miR-92a-3p | SLC12A5 | 1 | No | 0 | 0 | 0 | |
| miR-92a-3p | FBXO33 | 0.975 | No | 0 | 0 | 0 | |
| miR-92a-3p | PTEN | 0.971 | Yes | 0 | 0 | 0 | |
| miR-92a-3p | NIPBL | 0.961 | No | 0 | 0 | 0 | |
| miR-92a-3p | PCDH9 | 0.952 | No | 0 | 0 | 0 | |
| miR-92a-3p | BAZ2B | 1 | Yes | 0 | 0 | 0 | |
| miR-92a-3p | SCN8A | 0.777 | Yes | 0 | 0 | 0 | |
| miR-92a-3p | SLC6A1 | 0.977 | No | 0 | 0 | 0 | |
| miR-92a-3p | MTF1 | 0.978 | Yes | 0 | 0 | 0 | |
| **MicroRNA** | **Gene** | **Target Score** | **Experiment Supported** | **SFARI**  **Gene score** | **Syndromic** | **Reports (#)** | |
| miR-92a-3p | SHOX | 0.992 | No | 0 | 0 | 0 |  |
| miR-92a-3p | SLC4A10 | 0.984 | No | 3 | 0 | 10 |  |
| miR-92a-3p | CIC | 1 | Yes | 4 | 0 | 1 |  |
| miR-410-3p | SLC30A5 | 0.972 | No | 0 | 0 | 0 |  |
| miR-410-3p | KAT6A | 0.958 | No | 0 | 0 | 0 |  |
| miR-410-3p | PTBP2 | 0.967 | No | 0 | 0 | 0 |  |
| miR-410-3p | CD38 | 0.974 | No | 0 | 0 | 0 |  |
| miR-410-3p | AFF4 | 0.96 | No | 0 | 0 | 0 |  |
| miR-410-3p | NDUFA5 | 0.972 | No | 0 | 0 | 0 |  |
| miR-410-3p | FMR1 | 0.993 | No | 0 | 0 | 0 |  |
| miR-410-3p | PCDH10 | 0.994 | No | 0 | 0 | 0 |  |
| miR-410-3p | CNKSR2 | 0.96 | No | 0 | 0 | 0 |  |
| miR-410-3p | ZSWIM6 | 0.991 | No | 0 | 0 | 0 |  |
| miR-410-3p | PTPRC | 0.98 | No | 0 | 0 | 0 |  |
| miR-410-3p | AP1S2 | 0.951 | No | 0 | 0 | 0 |  |
| miR-410-3p | HCN1 | 0.954 | No | 0 | 0 | 0 |  |
| miR-410-3p | ATRX | 0.992 | No | 0 | 0 | 0 |  |
| miR-410-3p | RORA | 0.999 | No | 0 | 0 | 0 |  |
| miR-410-3p | RPS6KA3 | 0.971 | No | 0 | 0 | 0 |  |
| miR-410-3p | KAT2B | 0.954 | No | 0 | 0 | 0 |  |
| miR-410-3p | CTNNA3 | 0.967 | No | 0 | 0 | 0 |  |
| miR-410-3p | FOXP2 | 0.953 | No | 0 | 0 | 0 |  |
| miR-410-3p | GUCY1A2 | 1 | No | 0 | 0 | 0 |  |
| miR-410-3p | CNTN4 | 0.955 | No | 0 | 0 | 0 |  |
| miR-410-3p | CEP290 | 0.994 | No | 0 | 0 | 0 |  |
| miR-410-3p | FBXO33 | 0.997 | No | 0 | 0 | 0 |  |
| miR-410-3p | TBL1XR1 | 0.975 | No | 0 | 0 | 0 |  |
| miR-410-3p | BAZ2B | 0.989 | No | 0 | 0 | 0 |  |
| miR-410-3p | SOX5 | 0.959 | No | 0 | 0 | 0 |  |
| miR-3916 | NCKAP1 | 0.993 | No | 0 | 0 | 3 |  |
| miR-3916 | GPR139 | 0.992 | No | 0 | 0 | 3 |  |
| miR-3916 | GRIN2B | 0.961 | No | 0 | 0 | 2 |  |
| miR-3916 | SRGAP3 | 0.985 | No | 3 | 0 | 4 |  |
| miR-3916 | ATP8A1 | 0.986 | No | 3 | 0 | 3 |  |
| miR-3916 | CNR1 | 0.952 | No | 3 | 0 | 2 |  |
| miR-3916 | PCDHA11 | 0.95 | No | 4 | 0 | 5 |  |
| miR-3916 | FOXP1 | 0.965 | No | 4 | 0 | 2 |  |
| miR-3916 | AR | 0.989 | No | 4 | 0 | 2 |  |
| miR-3916 | PCDHA5 | 0.95 | No | 4 | 0 | 2 |  |
| miR-3916 | ROBO1 | 0.956 | No | 6 | 0 | 6 |  |
| **MicroRNA** | **Gene** | **Target Score** | **Experiment Supported** | **SFARI**  **Gene score** | **Syndromic** | **Reports (#)** |  |
| miR-378a-3p | SETD5 | 0.988 | No | 3 | 0 | 1 |  |
| miR-378a-3p | FOXG1 | 0.99 | No | 4 | 0 | 8 |  |
| miR-378a-3p | PTPN11 | 0.966 | No | 5 | 0 | 5 |  |
| miR-378a-3p | DSCAM | 0.985 | No | 5 | 0 | 5 |  |
| miR-361-5p | CSMD1 | 0.975 | No | 0 | 1 | 12 |  |
| miR-361-5p | MAP2 | 1 | No | 0 | 0 | 7 |  |
| miR-361-5p | CADM2 | 0.976 | No | 0 | 0 | 6 |  |
| miR-361-5p | ZNF804A | 0.987 | Yes | 0 | 0 | 4 |  |
| miR-361-5p | PRICKLE2 | 1 | No | 0 | 0 | 3 |  |
| miR-361-5p | RANBP17 | 0.998 | Yes | 0 | 1 | 1 |  |
| miR-361-5p | MET | 0.993 | Yes | 0 | 0 | 1 |  |
| miR-361-5p | RAC1 | 0.956 | No | 0 | 0 | 1 |  |
| miR-361-5p | PRKCB | 0.96 | No | 0 | 0 | 1 |  |
| miR-361-5p | NTNG1 | 0.974 | No | 3 | 1 | 25 |  |
| miR-361-5p | PARD3B | 0.986 | No | 3 | 0 | 2 |  |
| miR-361-5p | DPP10 | 0.99 | No | 4 | 0 | 15 |  |
| miR-361-5p | UBR5 | 0.992 | Yes | 4 | 0 | 4 |  |
| miR-361-5p | TBC1D31 | 0.965 | No | 4 | 0 | 3 |  |
| miR-361-5p | CREBBP | 0.976 | Yes | 4 | 0 | 3 |  |
| miR-361-5p | KMT2C | 0.98 | No | 4 | 0 | 2 |  |
| miR-361-5p | SLC24A2 | 0.966 | No | 5 | 0 | 8 |  |
| miR-146b-5p | CASK | 0.972 | Yes | 0 | 1 | 2 |  |
| miR-146b-5p | PHF3 | 0.967 | Yes | 3 | 0 | 26 |  |
| miR-146b-5p | ERBB4 | 0.977 | No | 4 | 0 | 5 |  |
| miR-146b-3p | FMR1 | 0.969 | No | 0 | 0 | 0 |  |
| miR-146b-3p | CNTNAP2 | 0.989 | No | 0 | 0 | 0 |  |
| miR-146b-3p | DLGAP1 | 0.984 | No | 0 | 0 | 0 |  |
| miR-146b-3p | CUX1 | 0.981 | No | 0 | 0 | 0 |  |
| miR-146b-3p | DNMT3A | 0.959 | No | 0 | 0 | 0 |  |
| miR-146b-3p | ZSWIM5 | 0.96 | No | 0 | 0 | 0 |  |
| miR-146a-5p | CASK | 0.971 | Yes | 0 | 0 | 0 |  |
| miR-146a-5p | PHF3 | 0.961 | Yes | 0 | 0 | 0 |  |
| miR-146a-5p | DLGAP2 | 0.957 | No | 0 | 0 | 0 |  |
| miR-146a-5p | NOS1 | 0.955 | No | 0 | 0 | 0 |  |
| miR-146a-5p | ERBB4 | 0.976 | Yes | 0 | 0 | 0 |  |
| miR-146a-3p | RANBP17 | 0.968 | No | 0 | 0 | 0 |  |
| miR-146a-3p | FOLH1 | 0.983 | No | 0 | 0 | 0 |  |
| miR-146a-3p | FOXP2 | 0.997 | No | 0 | 0 | 0 |  |
| miR-146a-3p | ZNF462 | 0.965 | No | 0 | 0 | 0 |  |
| miR-125a-5p | SLC4A10 | 0.995 | No | 0 | 1 | 6 |  |
| miR-125a-5p | NRXN1 | 0.959 | No | 0 | 0 | 2 |  |
| **MicroRNA** | **Gene** | **Target Score** | **Experiment Supported** | **SFARI**  **Gene score** | **Syndromic** | **Reports (#)** |  |
| miR-125a-5p | ZSWIM6 | 1 | Yes | 2 | 0 | 4 |  |
| miR-125a-5p | TET2 | 0.968 | Yes | 3 | 0 | 1 |  |
| miR-125a-5p | DIP2A | 0.964 | Yes | 4 | 0 | 8 |  |
| miR-125a-5p | LRFN2 | 0.997 | No | 4 | 0 | 8 |  |
| miR-125a-5p | ZNF827 | 0.979 | No | 4 | 0 | 7 |  |
| miR-125a-5p | CTTNBP2 | 0.986 | No | 4 | 0 | 4 |  |
| miR-125a-5p | ZSWIM5 | 1 | No | 4 | 0 | 3 |  |
| miR-125a-5p | RORA | 0.98 | Yes | 4 | 0 | 1 |  |
| miR-125a-5p | KLC2 | 0.989 | Yes | 5 | 0 | 2 |  |
| miR-125a-5p | IL16 | 0.982 | No | 5 | 0 | 1 |  |
| miR-125a-5p | TMLHE | 0.951 | No | 5 | 0 | 1 |  |
| miR-10a-5p | JARID2 | 0.996 | Yes | 0 | 0 | 0 |  |
| miR-10a-5p | ZNF827 | 0.973 | No | 0 | 0 | 0 |  |
| miR-10a-5p | RB1CC1 | 0.977 | Yes | 0 | 0 | 0 |  |
| miR-10a-5p | CADM2 | 0.993 | No | 0 | 0 | 0 |  |
| miR-10a-5p | ELAVL2 | 0.968 | No | 0 | 0 | 0 |  |
| miR-10a-5p | PCDH10 | 0.998 | No | 0 | 0 | 0 |  |
| miR-10a-5p | BDNF | 0.996 | No | 0 | 0 | 0 |  |
| miR-10a-5p | HCN1 | 1 | No | 0 | 0 | 0 |  |
| miR-10a-5p | RORA | 1 | Yes | 0 | 0 | 0 |  |
| miR-10a-5p | MDGA2 | 0.999 | No | 0 | 0 | 0 |  |
| miR-10a-5p | CAMK2B | 0.975 | No | 0 | 0 | 0 |  |
| miR-10a-3p | RBM27 | 0.96 | No | 0 | 0 | 0 |  |
| miR-10a-3p | MIB1 | 0.958 | No | 0 | 0 | 0 |  |
| miR-10a-3p | GUCY1A2 | 0.973 | No | 0 | 0 | 0 |  |
| miR-10a-3p | PTEN | 0.985 | No | 0 | 0 | 0 |  |
| miR-106a-5p | ESR1 | 0.996 | No | 0 | 1 | 10 |  |
| miR-106a-5p | VLDLR | 0.999 | Yes | 0 | 1 | 7 |  |
| miR-106a-5p | KATNAL1 | 0.976 | Yes | 0 | 1 | 6 |  |
| miR-106a-5p | PCDHA4 | 0.995 | No | 0 | 0 | 5 |  |
| miR-106a-5p | BRAF | 0.956 | No | 0 | 1 | 4 |  |
| miR-106a-5p | UBR5 | 0.991 | Yes | 0 | 1 | 2 |  |
| miR-106a-5p | ELAVL2 | 0.974 | Yes | 0 | 1 | 1 |  |
| miR-106a-5p | MTF1 | 0.959 | Yes | 0 | 0 | 1 |  |
| miR-106a-5p | ATG7 | 0.985 | No | 1 | 1 | 21 |  |
| miR-106a-5p | RLIM | 0.978 | Yes | 2 | 1 | 66 |  |
| miR-106a-5p | PCDHA13 | 0.995 | No | 2 | 1 | 34 |  |
| miR-106a-5p | SIK1 | 0.974 | Yes | 2 | 0 | 20 |  |
| miR-106a-5p | PCDHA3 | 0.995 | No | 2 | 0 | 20 |  |
| miR-106a-5p | MKL2 | 0.957 | No | 2 | 1 | 10 |  |
| miR-106a-5p | WDFY3 | 0.962 | No | 2 | 0 | 5 |  |
| **MicroRNA** | **Gene** | **Target Score** | **Experiment Supported** | **SFARI**  **Gene score** | **Syndromic** | **Reports (#)** |  |
| miR-106a-5p | DCUN1D1 | 0.976 | Yes | 2 | 0 | 4 |  |
| miR-106a-5p | ST8SIA2 | 0.973 | No | 3 | 0 | 17 |  |
| miR-106a-5p | PCDHA8 | 0.995 | No | 3 | 0 | 16 |  |
| miR-106a-5p | PCDHAC1 | 0.994 | No | 3 | 0 | 14 |  |
| miR-106a-5p | ZNF827 | 0.998 | Yes | 3 | 0 | 12 |  |
| miR-106a-5p | SLC25A27 | 0.985 | No | 3 | 0 | 12 |  |
| miR-106a-5p | NBEA | 0.98 | No | 3 | 0 | 10 |  |
| miR-106a-5p | SDC2 | 0.966 | No | 3 | 0 | 7 |  |
| miR-106a-5p | SCN2A | 0.954 | No | 3 | 0 | 3 |  |
| miR-106a-5p | KCNB1 | 0.969 | Yes | 3 | 0 | 2 |  |
| miR-106a-5p | RAB11FIP5 | 0.998 | Yes | 3 | 0 | 2 |  |
| miR-106a-5p | PCDHA5 | 0.995 | No | 3 | 0 | 2 |  |
| miR-106a-5p | CAMTA1 | 0.985 | Yes | 4 | 0 | 17 |  |
| miR-106a-5p | PCDHA6 | 0.995 | No | 4 | 0 | 15 |  |
| miR-106a-5p | PCDHA10 | 0.995 | No | 4 | 0 | 14 |  |
| miR-106a-5p | HTR2A | 0.956 | No | 4 | 0 | 13 |  |
| miR-106a-5p | PCDHA1 | 0.995 | No | 4 | 0 | 11 |  |
| miR-106a-5p | PCDHA12 | 0.995 | No | 4 | 0 | 8 |  |
| miR-106a-5p | KAT2B | 0.973 | No | 4 | 0 | 8 |  |
| miR-106a-5p | KMT2A | 0.97 | No | 4 | 0 | 8 |  |
| miR-106a-5p | DOCK4 | 0.994 | Yes | 4 | 0 | 7 |  |
| miR-106a-5p | SLC33A1 | 0.954 | No | 4 | 0 | 6 |  |
| miR-106a-5p | MYT1L | 0.998 | No | 4 | 0 | 5 |  |
| miR-106a-5p | FOXP1 | 0.954 | No | 4 | 0 | 4 |  |
| miR-106a-5p | PCDHAC2 | 0.993 | No | 4 | 0 | 3 |  |
| miR-106a-5p | UNC80 | 0.985 | No | 4 | 0 | 3 |  |
| miR-106a-5p | RPS6KA2 | 0.962 | Yes | 4 | 1 | 3 |  |
| miR-106a-5p | PCDHA2 | 0.995 | No | 4 | 0 | 2 |  |
| miR-106a-5p | TNRC6B | 0.991 | Yes | 4 | 0 | 1 |  |
| miR-106a-5p | RORA | 0.996 | Yes | 4 | 0 | 1 |  |
| miR-106a-5p | PCDHA11 | 0.995 | No | 5 | 0 | 10 |  |
| miR-106a-5p | SCN1A | 0.96 | No | 5 | 0 | 8 |  |
| miR-106a-5p | GLO1 | 0.951 | Yes | 5 | 0 | 4 |  |
| miR-106a-5p | WDR26 | 0.953 | Yes | 5 | 0 | 2 |  |
| miR-106a-5p | PCDHA7 | 0.995 | No | 5 | 0 | 1 |  |
| miR-106a-5p | GUCY1A2 | 0.971 | No | 5 | 0 | 1 |  |

For each microRNA-gene pair, the micro-T-cds target score denotes predicted strength of interaction (where 1.0 is the strongest possible interaction). Experimentally validated interactions are noted. Confidence of ASD association is noted for each gene on a 0-6 scale, based on the Simons Foundation Autism Research Initiative (SFARI; <https://www.sfari.org/resource/sfari-gene/>) algorithm. Gene involvement in syndromic cases and number of previous study reports are taken directly from the SFARI database.
