# Supplementary material for: Validation of a Salivary RNA Test for Childhood Autism Spectrum Disorder
Source: Front Genet. 2018 Nov 9;9:534. doi: 10.3389/fgene.2018.00534 (PMC6237842; doi:10.3389/fgene.2018.00534)
Supplement: TABLE S4 — KEGG Pathways over-represented by microRNA targets. The KEGG pathways over-represented (FDR < 0.05) by high-confidence gene targets of the microRNA classifiers are listed. The number of genes targeted within each pathway are shown along with the number of microRNAs targeting them. Note that several brain-related pathways have enriched numbers of targets, including axon guidance, neurotrophin signaling, and glioma. [file Table_4.docx]

**Additional Table 4. KEGG Pathways over-represented by microRNA targets**

| **KEGG pathway** | **FDR** | **Genes (#)** | **MicroRNAs (#)** | **MicroRNAs** |
| --- | --- | --- | --- | --- |
| Prion diseases | 0.0001 | 3 | 2 | miR-410, miR-146b |
| Axon guidance | 0.0005 | 26 | 8 | miR-106a-5p, miR-361-5p, miR-3916, miR-410, miR-92a-3p, miR-10a, miR-125a-5p, miR-146b |
| Proteoglycans in cancer | 0.0236 | 30 | 8 | miR-146b-5p, miR-146a, miR-92a-3p, miR-361-5p, miR-106a-5p, miR-10a, miR-410, miR-378a-3p |
| MAPK signaling pathway | 0.0236 | 37 | 11 | miR-410, miR-361-5p, miR-106a-5p, miR-92a-3p, miR-378a-3p, miR-10a, miR-125a-5p, miR-146b, miR-146b-5p, miR-146a, miR-10a-5p |
| mTOR signaling pathway | 0.0277 | 14 | 5 | miR-92a-3p, miR-10a, miR-106a-5p, miR-410, miR-361-5p |
| Neurotrophin signaling | 0.0277 | 21 | 9 | miR-10a, miR-378a, miR-106a-5p, miR-410, miR-146b, miR-92a-3p, miR-146b-5p, miR-361-5p |
| Ras signaling pathway | 0.0341 | 31 | 11 | miR-378a-3p, miR-361-5p, miR-92a-3p, miR-106a-5p, miR-410, miR-125a-5p, miR-3916, miR-146b-5p, miR-146a, miR-10a, miR-146b |
| Renal cell carcinoma | 0.0341 | 15 | 6 | miR-106a-5p, miR-92a-3p, miR-361-5p, miR-410, miR-146b, miR-378a-3p |
| Glioma | 0.0347 | 11 | 5 | miR-106a-5p, miR-361-5p, miR-92a-3p, miR-10a-3p, miR-10a-5p |
| Long-term potentiation | 0.0347 | 14 | 6 | miR-106a-5p, miR-92a-3p, miR-361-5p, miR-410, miR-3916, miR-10a |
| Hippo signaling pathway | 0.0347 | 18 | 8 | miR-410, miR-92a-3p, miR-106a-5p, miR-378a-3p, miR-10a, miR-361-5p, miR-125a-5p, miR-146b |
| Circadian entrainment | 0.0389 | 17 | 9 | miR-92a-3p, miR-3916, miR-361-5p, miR-106a-5p, miR-10a, miR-410, miR-10a, miR-146a, miR-125a-5p |

Abbreviations: Kyoto Encyclopedia of Genes and Genomes (KEGG)
