# Supplementary material for: Validation of a Salivary RNA Test for Childhood Autism Spectrum Disorder
Source: Front Genet. 2018 Nov 9;9:534. doi: 10.3389/fgene.2018.00534 (PMC6237842; doi:10.3389/fgene.2018.00534)
Supplement: TABLE S5 — Pathway interactions among putative microRNA targets. DIANA miRPATH software was used to identify KEGG pathways whose gene components had putative interacting relationships with the 11 microRNA classifiers. Notably, several pathways involved in metabolism and immune pathways previously implicated in autism were on the list. [file Table_5.docx]

Additional Table 5. Pathway interactions among putative microRNA targets

| **KEGG pathway** | **FDR** | **Genes (#)** | **Genes** | **miRNAs (#)** | **miRNAs** |
| --- | --- | --- | --- | --- | --- |
| Prion diseases | 1.0E-25 | 2 | NCAM2, PRNP | 1 | miR-410 |
| Metabolism of xenobiotics by cytochrome P450 | 3.2E-08 | 3 | GSTO2, GSTA2, GSTM2 | 2 | miR-125a-5p, miR-361-5p |
| NF-kappa B signaling pathway | 0.0004 | 2 | TRAF6, IRAK1 | 2 | miR-146b, miR-146a |
| ECM-receptor interaction | 0.0222 | 5 | COL24A1, COL27A1, ITGA5, ITGAV, COL1A2 | 2 | miR-378a-3p, miR-92a-3p |
| Glycosphingolipid biosynthesis - lacto and neolacto series | 0.0418 | 1 | FUT4 | 1 | miR-125a-5p |
| Toll-like receptor signaling pathway | 0.0421 | 3 | CD80, TRAF6, IRAK1 | 2 | miR-146b, miR-146a |

Abbreviations: Kyoto Encyclopedia of Genes and Genomes (KEGG)
